# CHIP promotes the activation of NF-κB signaling through enhancing the K63-linked ubiquitination of TAK1

**DOI:** 10.1038/s41420-021-00637-3

**Published:** 2021-09-17

**Authors:** Yuchun Liu, Yao Sun, Shaoming Han, Yanan Guo, Qingnan Tian, Qiang Ma, Shoutao Zhang

**Affiliations:** 1 School of Life Sciences, Zhengzhou University, Zhengzhou, Henan China; 2 Henan Neurodevelopment Engineering Research Center for Children, Children’s Hospital Affiliated to Zhengzhou University, Henan Children’s Hospital, Zhengzhou Children’s Hospital, Zhengzhou, Henan China

**Keywords:** Cell biology, Innate immunity

## Abstract

Transcriptional factor nuclear factor κB (NF-κB) can be activated by various intracellular or extracellular stimuli and its dysregulation leads to pathological conditions, such as neurodegenerative disorders, infection, and cancer. The carboxyl terminus of HSC70-interacting protein (CHIP), a pathogenic gene of spinocerebellar autosomal recessive 16 (SCAR16), plays an important roles in protein degradation, trafficking, and multiple signaling transductions. It has been reported that CHIP participates in the regulation of NF-κB signaling, and the mutant of CHIP (p.T246M) leads to the occurrence of SCAR16. However, the detailed mechanism of CHIP and CHIP (p.T246M) in the regulation of NF-κB signaling in neurological disorders remains unclear. Here, we found that CHIP promoted the activation of NF-κB signaling, while the knockdown had the opposite effect. Furthermore, CHIP interacted with TAK1 and targeted it for K63-linked ubiquitination. Finally, CHIP enhanced the interaction between TAK1 and NEMO. However, CHIP (p.T246M) couldn’t upregulate NF-κB signaling, potentiate the ubiquitination of TAK1, and enhance the interactions. Taken together, our study demonstrated for the first time that CHIP positively regulates NF-κB signaling by targeting TAK1 and enhancing its K63-linked ubiquitination.

## Introduction

The nuclear factor κB (NF-κB) plays a crucial role in cell proliferation, apoptosis, immunity, and inflammation [1]. The NF-κB signaling pathway, initially characterized as the context of the immune system, involves in many other diseases like neurodegenerative disorders, cancers, and metabolic diseases [2–5]. In the unstimulated cells, nuclear factor κB interacts with the inhibitory protein of NF-κB (IκBα), and retains in the cytoplasm. Upon the detection of stimuli, like TNFα, IL-1β, TLRs, proteotoxic, oxidative, and endoplasmic reticulum stress, NF-κB signaling is activated which leads to inflammation, proliferation, death, and apoptosis, while the detailed regulatory mechanisms of NF-κB signaling are still not well illuminated.

Ubiquitination, an important post-translational modification, has been implicated in NF-κB signaling [6, 7]. In the stimulated cells, cytokines are binding to signaling intermediaries, such as TNF receptor, IL-1 receptor, and Toll-like receptor, leading to the activation of TAK1 complex. The active TAK1 phosphorylates TGF-β-activated kinase 1 and IKK complexes (composed of IKKα, IKKβ, and NEMO), which further initiate NF-κB cascades. Next, the IKK complex becomes activated and phosphorylates IκBα. The activation of NF-κB finally occurs when IκBα is targeted for degradation and frees NF-κB for nuclear importing [8]. In this cascade, the subunits of TAK1 complex, IKK complex, and IκBα undergo different types of ubiquitination. The K63-linked ubiquitination of TAK1 and NEMO are known to regulate the activation of NF-κB signaling [9, 10]. The linear ubiquitination of NEMO is associated with its kinase activity [11]. IκBα is targeted for degradation after modification with K48-linked ubiquitination [12].

The carboxyl terminus of HSC70-interacting protein (CHIP), encoded by STUB1 gene, is an E3 ubiquitin ligase containing a three tetratricopeptide repeats domain and an U-box domain. The U-box domain of CHIP displays E3 ubiquitination ligase activity involving the processes of protein degradation, cell proliferation, and tumor progression [13]. The abnormal function of CHIP caused the spinocerebellar autosomal recessive 16 (SCAR16), a form of neurodegenerative disorders [14, 15]. It has been reported that CHIP participated in the regulation of NF-κB signaling to cancer development, osteoclast formation, and innate immunity [16–18]. However, the mechanism of CHIP in the regulation of NF-κB signaling in neurological disorders has not been illustrated.

In this study, we found that CHIP positively regulated NF-κB signaling. CHIP targeted TAK1 and promoted the K63-linked ubiquitination of TAK1 through the U-box domain. CHIP further enhanced the interaction of TAK1 and NEMO. While CHIP (p.T246M) could not activate NF-κB signaling as CHIP, knockdown of CHIP reduced the interaction between TAK1 and NEMO, and inhibited NF-κB activation. To our knowledge, our results revealed that CHIP was a positive regulator in NF-κB signaling, which will help to gain insight into neurological disorders.

## Results

### CHIP promotes the activation of TNFα-induced NF-κB signaling

**Fig. 1:**
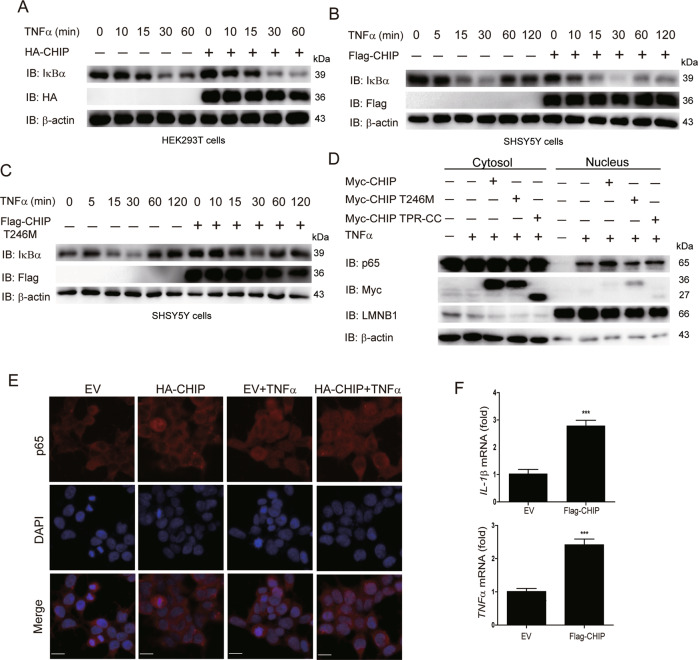
CHIP promotes the activation of TNFα-induced NF-κB signaling. **A**, **B** CHIP promoted the degradation of endogenous IκBα. HEK293T cells (**A**) or SHSY5Y cells (**B**) were transfected with an empty vector, HA-CHIP, or Flag-CHIP, and stimulated with TNFa (5 ng/ml) for 0, 5, 10, 15, 30, 60, or 120 min. **C** CHIP (p.T246M) could not degrade IκBα as CHIP. SHSY5Y cells were transfected with an empty vector or Flag-CHIP T246M, and stimulated with TNFa (5 ng/ml) for 0, 5, 15, 30, 60, or 120 min. **D** CHIP accelerated the nucleus translocation of p65. HEK293T cells were transfected with an empty vector, Myc-CHIP, Myc-CHIP T246M, or Myc-CHIP TPR-CC, and stimulated with TNFa (5 ng/ml) for 1 h. Western blot analyzed the endogenous p65 from cytosolic and nuclear fractions. **E** CHIP promoted the nuclear translocation of p65. HEK293T cells were transfected with an empty vector or HA-CHIP, and stimulated with TNFa (5 ng/ml) for 1 h, and then subjected to immunofluorescence analysis using a p65-specific antibody. DNA was visualized with DAPI (blue). Scale bar: 20 μm. **F** HEK293T cells were transfected with an empty vector or Flag-CHIP, and stimulated with TNFa (5 ng/ml) for 8 h. NF-κB signaling target genes (*IL-1β* and *TNFα*) expression detecting by quantitative real-time PCR. All results were representative of three independent experiments. The graphs showed the means ± SD of three independent experiments. *P < 0.05, **P < 0.01, ***P < 0.001, and ns no significant (Student’s *t*-test).

TNFα, producing by the activation of NF-κB signaling, is one of the important stimuli of NF-κB signaling which regulates inflammation, innate immune, cancers, and neurodegenerative diseases [19]. E3 ligases mediated ubiquitination in modification of post-transcription plays a vital role in the regulation of TNFα-induced NF-κB signaling [20]. Recent studies have reported that CHIP (p.T246M) mutant (CHIP is an E3 ubiquitin ligase) results in the occurrence of neurodegenerative disorders of SCAR16 [14]. To investigate the role of CHIP in the regulation of NF-κB signaling induced by TNFα, HEK293T, and SHSY5Y cells were transfected with CHIP, stimulated with TNFα, and monitored for the NF-κB activation by immunoblotting. As shown in Fig. 1A, B, CHIP promoted the degradation of endogenous IκBα in HEK293T and SHSY5Y cells. We also detected the function of CHIP (p.T246M) in NF-κB signaling, and found that CHIP (p.T246M) could not degrade IκBα as CHIP (Fig. 1C). Since the degradation of IκBα releases p65 for nuclear translocation and further activates the activation of NF-κB signaling, we next tested whether CHIP, CHIP (p.T246M), or CHIP TPR-CC affects the subcellular localization of p65 upon stimulating with TNFα. CHIP accelerated the nucleus translocation of p65, while CHIP (p.T246M) or CHIP TPR-CC didn’t have the function (Fig. 1D). We further detected the nuclear localization of p65 by immunofluorescence analysis, and found that CHIP promoted the nuclear translocation of p65 (Fig. 1E). Moreover, we found CHIP increased the expression levels of NF-κB target genes, such as *IL-1β* and *TNFα* by real-time PCR assays (Fig. 1F). Taken together, these data suggested that CHIP promoted the activation of TNFα-induced NF-κB signaling.

### Knockdown or knockout of CHIP inhibits NF-κB activation

**Fig. 2:**
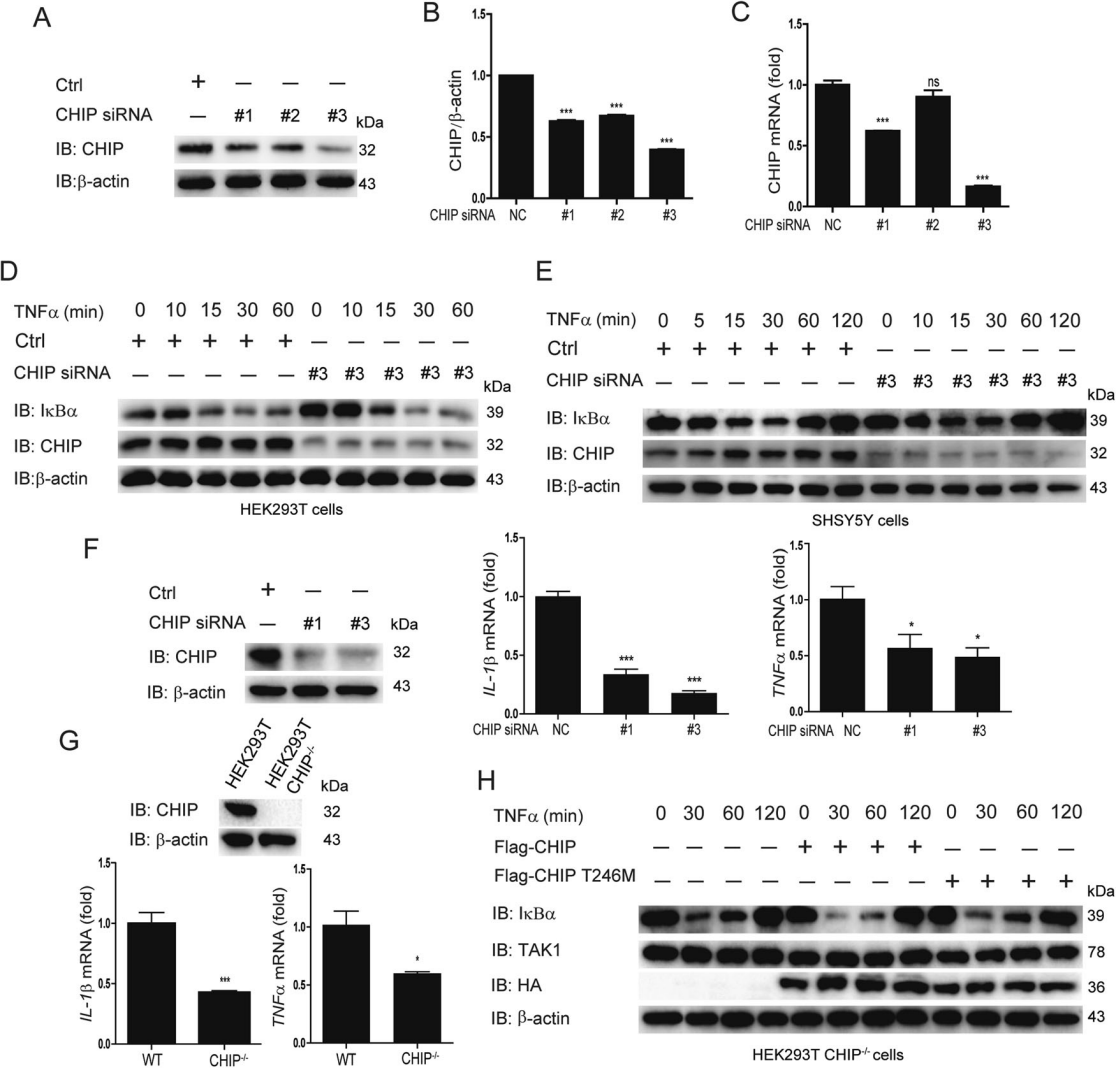
Knockdown or knockout of CHIP inhibits NF-κB activation. **A**–**C** The knockdown efficiency of CHIP-specific siRNAs were detected in protein level (**A**) and mRNA level (**C**), and the protein level of CHIP was quantified by Image J (**B**). HEK293T cells were transfected with control (NC) siRNA or CHIP-specific siRNA, and the knockdown was confirmed by western blot and quantitative real-time PCR. **D**, **E** Knockdown of CHIP inhibited the degradation of endogenous IκBα. HEK293T cells (**D**) or SHSY5Y cells (**E**) were transfected with control (NC) siRNA or CHIP-specific siRNA, and stimulated with TNFa (5 ng/ml) for 0, 5, 10, 15, 30, 60, or 120 min. **F**, **G** Knockdown or knockout of CHIP suppressed TNFα-induced NF-κB signaling. HEK293T cells were transfected with control (NC) siRNA or CHIP-specific siRNA, and stimulated with TNFa (5 ng/ml) for 8 h. Detecting the expression of NF-κB signaling target genes (*IL-1β* and *TNFα*) by quantitative real-time PCR, and the knockdown efficiency of CHIP-specific siRNAs by immunoblotting (**F**). *IL-1β* and *TNFα* expression were also analyzed in WT and CHIP^-/-^ HEK293T cells by quantitative real-time PCR, and the knockout of CHIP were detected by immunoblotting (**G**). **H** Re-introduction of CHIP promoted TNFα-induced NF-κB signaling, while CHIP T246M barely affected the activation of NF-κB signaling. CHIP^−/−^ HEK293T cells were transfected with an empty vector, Myc-CHIP, or Myc-CHIP T246M, and stimulated with TNFa (5 ng/ml) for indicated times. The cell lysates were detected by immunoblotting with indicated antibodies. All results were representative of three independent experiments. The graphs showed the means ± SD of three independent experiments. *P < 0.05, **P < 0.01, ***P < 0.001, and ns no significant (Student’s *t*-test).

Next, to detect whether endogenous CHIP was involved in the NF-κB signaling, we generated three pairs of siRNAs specific for CHIP, two of which (#1 and #3) validly inhibited the expression of CHIP at protein and mRNA levels (Fig. 2A–C). Immunoblotting results revealed that knockdown of CHIP significantly reduced the degradation of IκBα with the treatment with TNFα in HEK293T and SHSY5Y cells (Fig. 2D, E). Furthermore, we also obtained one CHIP^−/−^ HEK293T cell line using the CRISPR/Cas9 system. Knockdown or knockout of CHIP reduced the mRNA levels of *IL-1β* and *TNFα* by real-time PCR assays (Fig. 2F, G). When we re-introduced CHIP or CHIP (p.T246M) in CHIP^−/−^ HEK293T cell line, CHIP could promoted the activation of NF-κB signaling, while CHIP (p.T246M) didn’t have this function (Fig. 2H). Hence, these results indicated that knockdown of endogenous CHIP inhibits NF-κB activity.

### CHIP interacts with TAK1

**Fig. 3:**
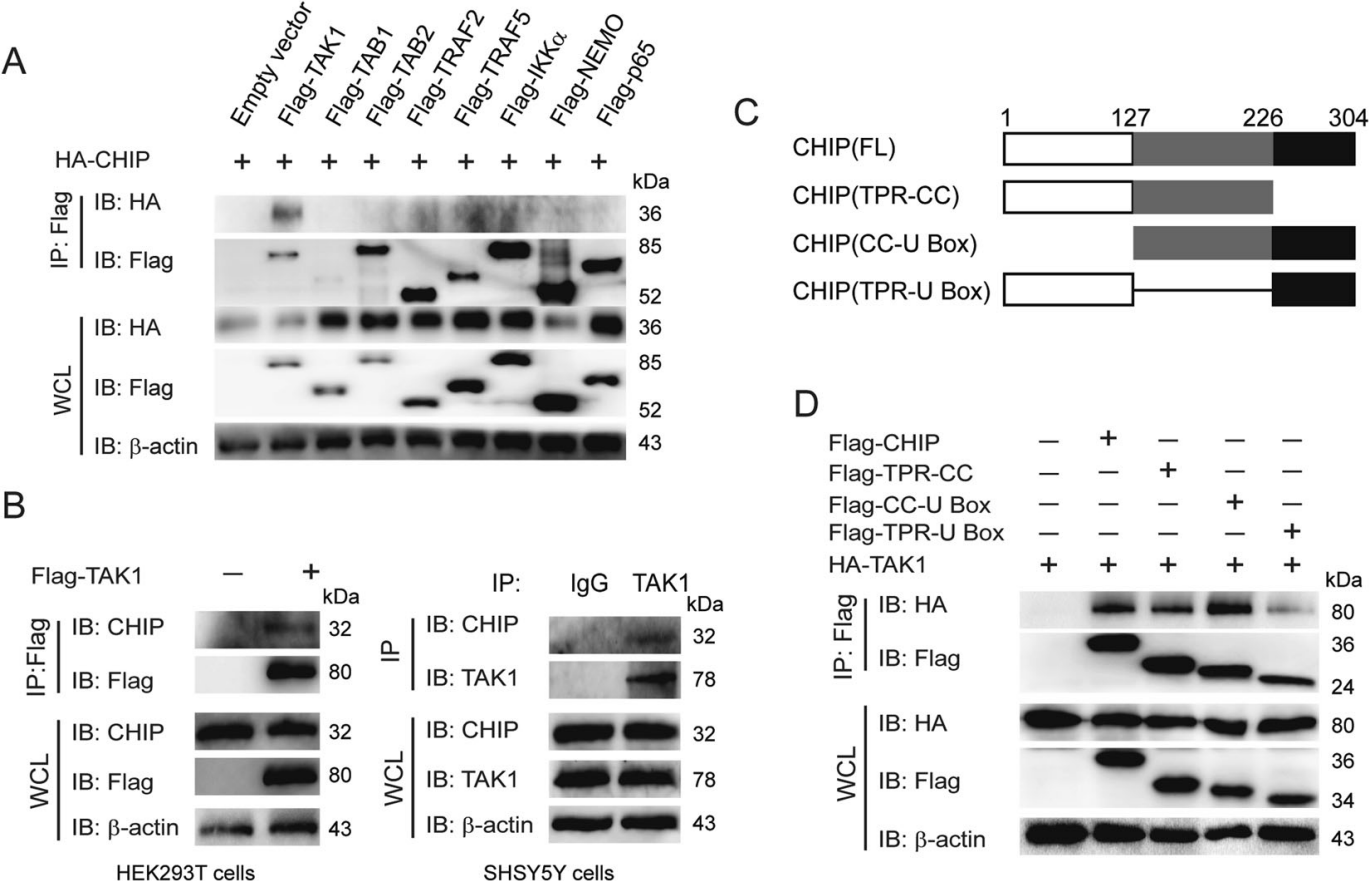
CHIP interacts with TAK1. **A** CHIP interacts with TAK1. HEK293T cells were transfected with HA-CHIP and an empty vector, Flag-TAK1, Flag-TAB1, Flag-TAB2, Flag-TRAF2, Flag-TRAF5, Flag-IKKα, Flag-NEMO, or Flag-p65, and the cell lysates were detected by co-immunoprecipitation and immunoblotting. **B** CHIP interacts with TAK1. Overexpression of TAK1 or endogenous TAK1 interacted with endogenous CHIP detecting by co-immunoprecipitation and immunoblotting in HEK293T or SHSY5Y cells. **C** The domain architecture of CHIP showing different deletion constructs of CHIP. Numbers indicated the amino acid position in construct. **D** HEK293T cells were transfected with HA-TAK1 and an empty vector, Flag-CHIP or Flag-CHIP domains. The cell lysates were detected by co-immunoprecipitation and immunoblotting. All results were representative of three independent experiments.

Previous studies found that CHIP can target TRAF2, TRAF6, or p65 for the regulation of NF-κB signaling [16, 17, 21]. TRAF6 is independent of the signaling cascade of TNFα-induced NF-κB pathway. So we detect the interaction between CHIP and downstream proteins of TNFα-induced NF-κB signaling, such as TAK1, TAB1, TAB2, TRAF2, TRAF5, IKKα, NEMO, and p65. Inconsistent with previous reports, we could not detect the obvious interactions between CHIP and TRAF2 or p65. Co-immunoprecipitation experiments revealed that CHIP had strong interaction with TAK1 (Fig. 3A). To determine the interaction, we further confirmed the interaction between TAK1 and endogenous CHIP, as well as endogenous interaction between TAK1 and CHIP. As shown in Fig. 3B, CHIP had the endogenous interaction with TAK1. We further detected which domains of CHIP were responsible for the interaction through generating three CHIP deletion mutant proteins (Fig. 3C). CHIP could not interact with TAK1 after deleting the CC domain which located between TPR and U box domains (Fig. 3D). It suggested that the CC domain of CHIP is essential for binding to TAK1. These results revealed that CHIP had an interaction with TAK1.

### CHIP enhanced the K63-linked ubiquitination

**Fig. 4:**
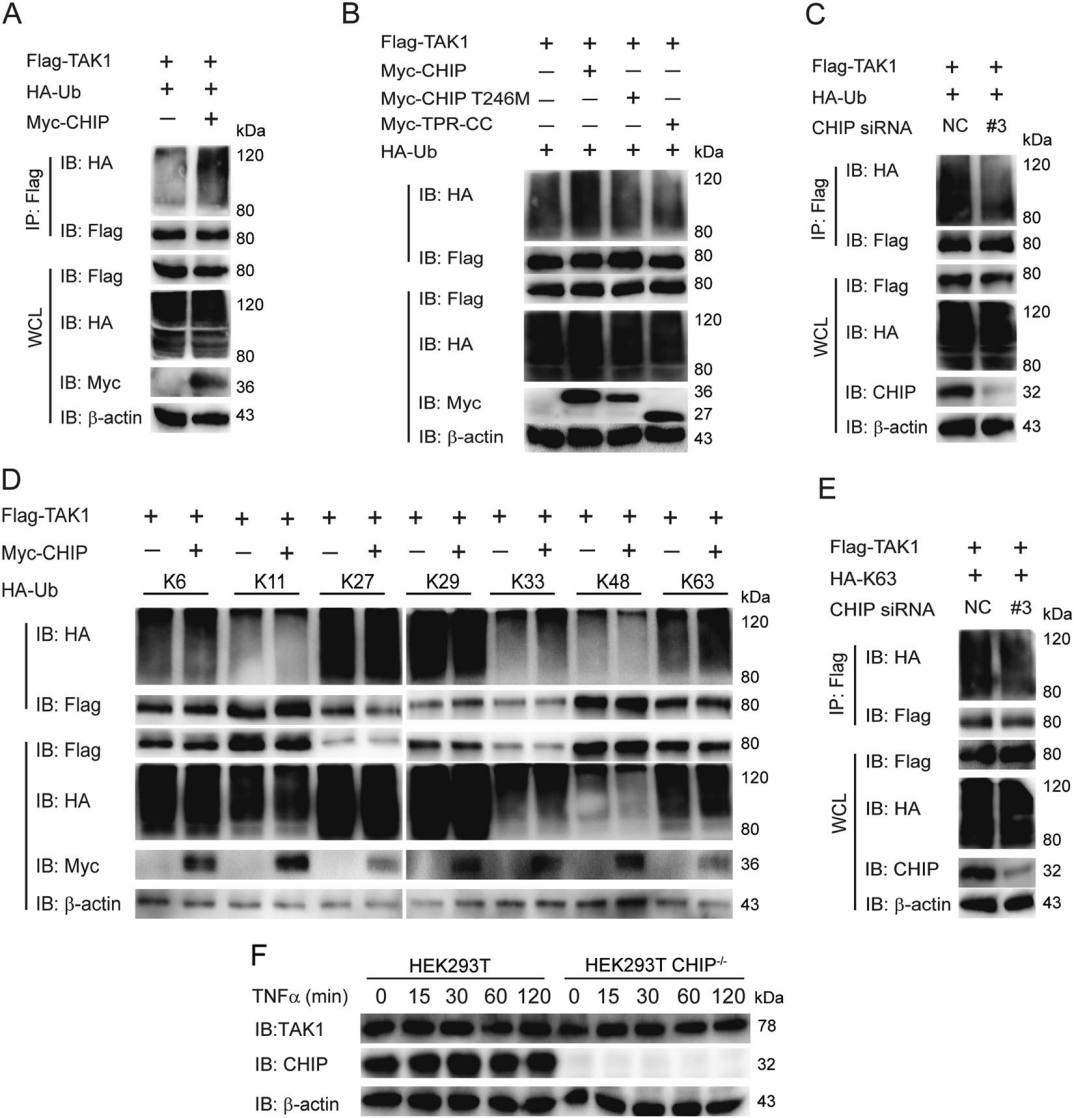
CHIP enhanced the K63-linked ubiquitination of TAK1. **A** Overexpression of CHIP increased the ubiquitination of TAK1. HEK293T cells were transfected with HA-tagged ubiquitin, Flag-TAK1, and an empty vector, or Myc-CHIP, Immunoprecipitation, and immunoblotting were performed to detect the ubiquitination of TAK1. **B** The inactive mutants of CHIP reduced the ubiquitination of TAK1. Immunoprecipitation and immunoblot analysis were performed to detect the ubiquitination of TAK1 after co-transfection with HA-tagged ubiquitin, Flag-TAK1, and an empty vector, Myc-CHIP T246M, Myc-TPR-CC, or Myc-CHIP in HEK293T cells. **C** Knockdown of CHIP reduced the ubiquitination of TAK1. HEK293T cells were transfected with HA-tagged ubiquitin, Flag-TAK1, and control siRNA (NC) or CHIP-specific siRNA, then the cell lysates were detected by co-immunoprecipitation and immunoblot. **D** Overexpression of CHIP promoted the K63-linked ubiquitination of TAK1. HEK293T cells were transfected with indicated plasmids as figures shown. Whole cell extracts were immunoprecipitated with anti-Flag beads and blotted with anti-HA antibody. **E** Knockdown of CHIP reduced the K63-linked ubiquitination of LEF1. Immunoprecipitation and immunoblot analysis were performed to detect the ubiquitination of TAK1 after co-transfection with HA-tagged K63-linked ubiquitin, Flag-TAK1, and control siRNA (NC) or CHIP-specific siRNA. **F** Knockout of CHIP didn’t influence the abundance of TAK1. WT and CHIP^−/−^ HEK293T cells were stimulated by TNFa (5 ng/ml) for indicated times. The cell lysates were detected by immunoblotting. All results were representative of three independent experiments.

CHIP, an E3 ligase enzyme, regulates the ubiquitination of many proteins like TRAF6, PTEN, LRRK2, and RIPK3 [17, 22–24]. So we also detect whether CHIP influences the ubiquitination of TAK1 by co-immunoprecipitation. We found that CHIP enhanced the ubiquitination of TAK1 (Fig. 4A). Since the U Box domain of CHIP is responsible for E3 ligase enzyme activity, and CHIP (p.T246M) is the ubiquitin enzymatically inactive mutant. So, we further test whether the U Box domain and CHIP (p.T246M) mutant affect the ubiquitination of TAK1.We observed that CHIP (p.T246M) mutant and CHIP TPR-CC could not enhance the ubiquitination of TAK1 (Fig. 4B). Moreover, knockdown of CHIP reduced the ubiquitination (Fig. 4C). At last, we examined which kinds of ubiquitination of TAK1 might be affected by CHIP. Overexpression of CHIP enhanced the K63-linked ubiquitination of TAK1, but had no effect on the K6, K11, K27, K29, K33, or K48-linked ubiquitination (Fig. 4D). Consistent with this result, knockdown of CHIP limited the K63-linked ubiquitination of TAK1 (Fig. 4E). To determine whether CHIP influenced the abundance of TAK1 through K63-linked ubiquitination, we further detected the endogenous TAK1 in WT and CHIP^−/−^ HEK293T cells, and found that CHIP didn’t influence the abundance of TAK1 (Fig. 4F). These results indicated that CHIP potentiated the K63-linked ubiquitination of TAK1.

### CHIP potentiates the interaction between TAK1 and NEMO

**Fig. 5:**
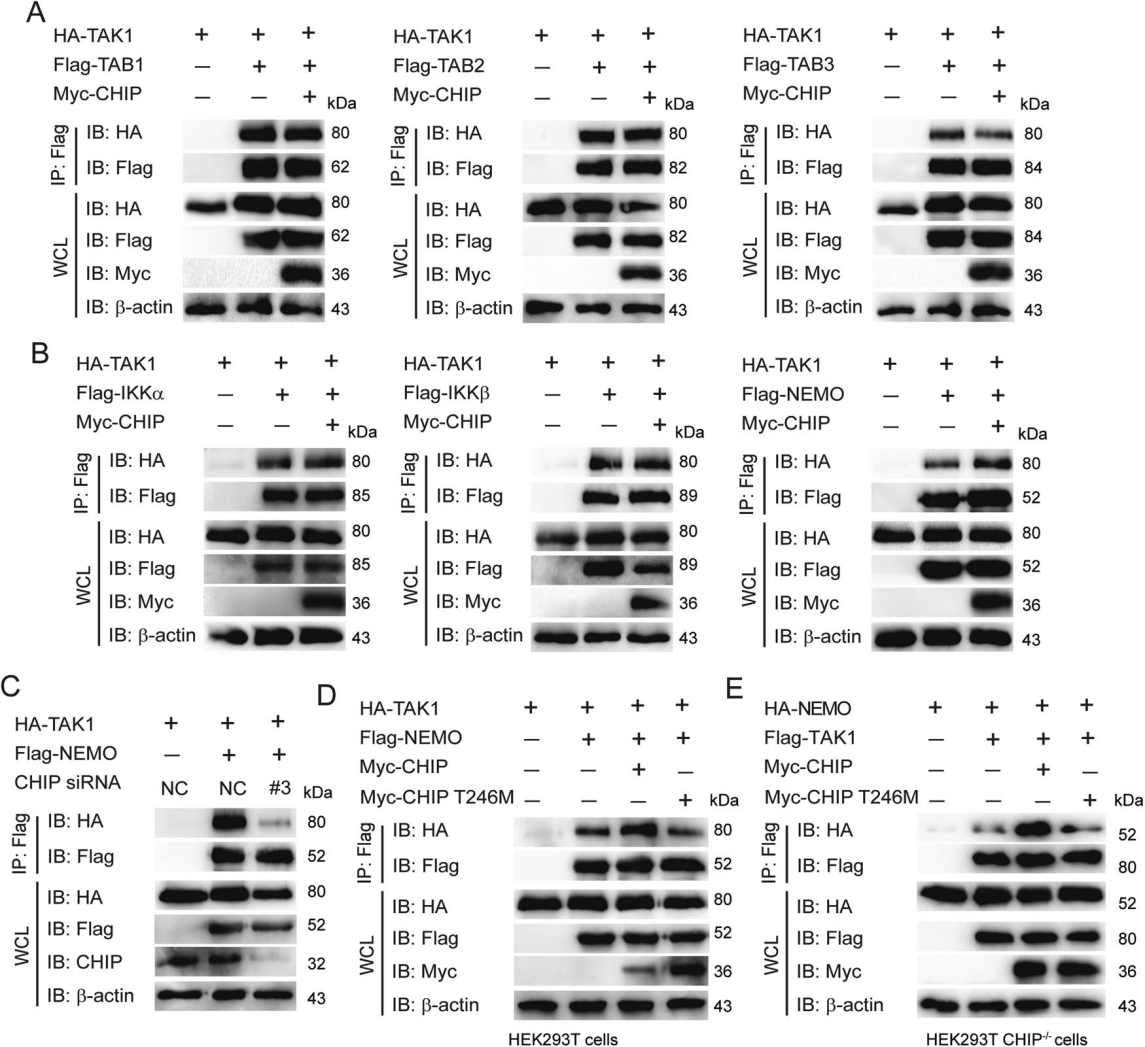
CHIP potentiates the interaction between TAK1 and NEMO. **A**, **B** Overexpression of CHIP enhanced the interaction between TAK1 and NEMO. Immunoprecipitation and immunoblot analysis of HEK293T cells which were transfected with indicated plasmids as figures shown. **C** Knockdown of CHIP reduced the interaction between TAK1 and NEMO. HEK293T cells were transfected with HA-TAK1, Flag-NEMO, and an empty vector or CHIP-specific siRNA. Whole cell extracts were immunoprecipitated with anti-Flag beads and blotted with anti-HA antibody. **D**, **E** The inactive mutant of CHIP could not enhance the interaction between TAK1 and NEMO. The indicated plasmids were transfected as figures shown in WT or CHIP^−/−^ HEK293T cells, and the cell lysates were performed to immunoprecipitation and immunoblot analysis. All results were representative of three independent experiments.

We have found that CHIP targeted and enhanced K63-linked ubiquitination of TAK1, and didn’t affect the degradation of TAK1. Therefore, we want to know whether CHIP influences the interaction between TAK1 and TAB or IKK complexes, which then promotes NF-κB signaling cascade. As shown in Fig. 5A, B, CHIP enhanced the interaction between TAK1 and NEMO, but had no effect on the interactions between CHIP and TAB1, TAB2, TAB3, IKKα, or IKKβ. Knockdown of CHIP decreased the interaction of CHIP-NEMO (Fig. 5C). Moreover, overexpression of CHIP T246M mutant couldn’t affect the interaction between TAK1 and NEMO in WT or CHIP^−/−^ HEK293T cells (Fig. 5D, E). These data suggested that CHIP enhanced the interaction between TAK1 and NEMO.

## Disscussion

NF-κB signaling regulates the transcription activity of multiple genes, which plays an important roles in inflammation, immune responses, cancer, and neurodegenerative disorders. Increasing evidence indicates that NF-κB signaling influences the occurrence and progression of Alzheimer’s disease, Parkinson’s disease, amyotrophic lateral sclerosis (ALS), and multiple sclerosis (MS) through regulating the neuroimmunological response [3, 25–27]. The deregulated NF-κB signaling may result in neurodegenerative diseases. An increasing number of E3 ubiquitin ligases and deubiquitinases play vital roles in the regulation of NF-κB signaling at the post-translational level of target proteins. TRIM13, TRIM25, TRIM38, USP18, and A20 were identified as regulators of NF-κB signaling by targeting and ubiquitination of TAK1, NEMO, TRAF6, TAB2/3, or TRAF2 [28–32]. The involvement of so many proteins in the regulation of NF-κB signaling suggests the importance of the complicated regulatory network to ensure an appropriate response to stimuli.

Recent studies indicated that the mutant of CHIP (p.T246M) lost E3 ligase activity, but maintained the features of the interactions with chaperones and chaperone-related functions, which further lead to the occurrence of SCAR16 [33]. And some reports have found that CHIP interacted with Tau, Parkin, or Ataxin-1 involving in the pathogenesis of various neurodegenerative diseases [34–36]. While the detailed mechanisms of CHIP (p.T246M) regulate the neurodegenerative diseases through NF-κB signaling are not clear. In this study, we found CHIP, an E3 ubiquitin ligase, participated in the upregulation of NF-κB signaling. Overexpression of CHIP promoted the degradation of endogenous IκBα in HEK293T and SHSY5Y cells, increased the nuclear translocation of p65, enhanced the transcription of *IL-1β* and *TNFα*. Knockdown of CHIP or the ubiquitin enzymatically inactive mutant CHIP (p.T246M) didn’t have the function. Re-introduction of CHIP WT into the CHIP^−/−^ HEK293T cells promoted the degradation of endogenous IκBα, while re-introduction of CHIP (p.T246M) mutant barely affected the activation of NF-κB signaling.

Previous studies found that CHIP is involved in cell proliferation, apoptosis, autophagy, cancer progression, and neurodegenerative diseases. CHIP regulated the ubiquitination of RIPK3, PTEN, and Smad1/Smad4 to influence necroptosis, autophagy, and TGF-β pathway [22, 24, 37]. CHIP also regulates NF-κB signaling, which leads to the occurrence of several diseases. For example, CHIP negatively regulated NF-κB signaling by targeting p65 in colorectal cancer, degrading TRAF6 in bone associated diseases, and targeting TRAF2 in breast cancer [16, 17, 21]. CHIP facilitated TLR-induced NF-κB signaling by recruiting Src and atypical PKCζ in the mature of macrophages and DCs [18]. Our data indicated the CHIP promoted TNFα-induced NF-κB signaling by targeting TAK1. Exogenous and endogenous immunoprecipitation revealed that CHIP interacted with TAK1, but not TAB1, TAB2, TRAF2, TRAF5, IKKα, NEMO, or p65. Detection the CC domain of CHIP, which located between TPR and U box domains could not interact with CHIP. CHIP further potentiated the ubiquitination of TAK1, particularly the K63-linked ubiquitination. The ubiquitin enzymatically inactive mutant CHIP (p.T246M) and CHIP TPR-CC couldn’t increase the ubiquitination of TAK1. Knockdown of CHIP inhibited the ubiquitination. Furthermore, the K63-linked ubiquitination of TAK1 which caused by CHIP didn’t affect the degradation of TAK1. Finally, we found that CHIP enhanced the interaction between TAK1 and NEMO, but not others. The interaction between TAK1 and NEMO, which influenced by CHIP or CHIP (p.T246M) was also confirmed in CHIP^−/−^ HEK293T cells. From this study, we also found CHIP could regulate the activation of Wnt signaling which might link the crosstalk between NF-κB and Wnt signaling.

In summary, our study revealed a novel regulatory mechanism that CHIP upregulates NF-κB signaling by targeting TAK1. CHIP, an E3 ligase, promotes the activation of TNFα-induced NF-κB signaling, interacts with TAK1, enhances the ubiquitination of TAK1, and further potentiates the interaction between TAK1 and NEMO. However, CHIP (p.T246M) can’t promote the activation of NF-κB signaling, and knockdown of CHIP further inhibits NF-κB signaling. Thus, these results may be helpful to realize the occurrence mechanisms of neurodegenerative diseases SCAR16.

## Materials and methods

### Reagents and plasmids

Anti-β-actin (A1978), horseradish peroxidase (HRP)-anti-Flag (M2) (A8592), anti-mouse-IgG-HRP (AP308P), and anti-rabbit-IgG-HRP (AP132P) were purchased from Sigma (St. Louis, MO, USA). HRP-anti-hemagglutinin (12013819001) was purchased from Roche Applied Science (Switzerland, Basel). Anti-c-Myc (HT101) was purchased from TransGen Biotech (Beijing, China). Anti-TAK1 (5206) and p65 (8242) were purchased from Cell Signaling Technology (Danvers, MA, USA). Anti-LMNB1 (101237-T32) and anti-STUB1 (12496-R034) were purchased from Sino Biological (Beijing, China). Anti-CHIP (sc-133066) was purchased from Santa Cruz Biotechnology (San Diego, CA, USA). TNFα Recombinant Human Protein (10602HNAE25) was purchased from Thermo Fisher (Waltham, MA, USA).

Empty vector pcDNA3.1 was kindly provided by Dr Jun Cui (Sun Yat-sen University, Guangzhou, China). Target genes were cloned from SHSY5Y cDNA and then subcloned into the pcDNA3.1 vector. The recombinant plasmids were confirmed by DNA sequencing in Sangon Biotech (Shanghai, China).

### Cell culture and transfection

Human embryonic kidney 293T (HEK293T) cells were kindly provided by Dr Jun Cui (Sun Yat-sen University, Guangzhou, China). CHIP^−/−^ HEK293T cell line was constructed and preserved by our lab. SHSY5Y cells were obtained from Cell Bank of the Chinese Academy of Sciences (Shanghai, China). These cells were cultured in DMEM (Hyclone, Logan, UT, USA) with 10% fetal bovine serum (BioIn, Israel), and 1% l-glutamine (Gibco, Carlsbad, CA, USA) at 37 °C in 5% CO_2_. The cells were transfected with expression plasmids by Lipofectamine 2000 (Invitrogen, Carlsbad, CA, USA) according to the manufacturer’s instructions.

### Immunoblot analysis

To monitor the protein turnover, HEK293T or SHSY5Y cells were plated in 24-well plates at a density of 5 × 10^4^ cells per well and transfected with indicated plasmids using Lipofectamine 2000 (Invitrogen). Lysates from the transfected cells were extracted with 120 μl low-salt lysis buffer (50 mM Hepes pH 7.5, 150 mM NaCl, 1 mM EDTA, 1.5 mM MgCl_2_, 10% glycerol, 1% Triton X-100), supplemented with 5 mg/ml protease inhibitor (Thermo) and phosphatase inhibitor Cocktail (Roche). The equal protein of each samples were loaded to SDS-PAGE and transferred onto PVDF membranes (Millipore, Schwalbach, Germany). Membranes were blocked using 5% skim milk (Solarbio, Beijing, China), incubated with specific antibodies, and subsequently detected using chemiluminescence (Millipore).

Cells were plated in 6-well plates at a density of 3 × 10^5^ cells per well and transfected with indicated plasmids using Lipofectamine 2000. Cytosolic and nuclear fractions of cells were separated using Minute^TM^ Cytoplasmic and Nuclear Fractionation Kit for Cells (Invent Biotechnologies, Eden Prairie, MN, USA) according to the manufacturer’s instructions.

### Immunoprecipitation analysis

To detect the protein–protein interaction, the immunoprecipitation experiments were performed. Whole cell lysates were prepared as the method of immunoblot assay, and incubated with the anti-Flag agarose gels (Sigma) on roller shaker overnight at 4 °C. The beads were washed six times with low salt lysis buffer, re-suspended with 2× SDS Loading Buffer (Solarbio), and boiled for 10 min. The released proteins were subjected to western blot analyses with the indicated antibodies.

For endogenous immunoprecipitation experiments, the extracted cell proteins were obtained as the method of immunoblot assay, and then incubated with indicated antibodies overnight at 4 °C. Twenty microliter Dynabeads protein G (Invitrogen) were added to the extracted cell proteins and incubated for another 2 h. Then the beads were washed and subjected to western analyses with the indicated antibodies.

### Real-time PCR

The cells were plated in 24-well plates at a density of 5 × 10^4^ cells per well and transfected with indicated plasmids using Lipofectamine 2000. Total RNA was isolated from cells by TRIzol Reagent (Invitrogen) according to the manufacturer’s protocol. The first strand cDNA was synthesized using HiScript III 1st Strand cDNA Synthesis Kit (+gDNA wiper) (Vazyme, Nanjing, China). Real-time PCR was performed with the ChamQ^TM^ Universal SYBR qPCR Master Mix (Vazyme). The cycling conditions were 94 °C for 5 min, followed by 40 cycles at 94 °C for 20 s, at 55 °C for 20 s, at 72 °C for 20 s. The specific primers are used for real-time PCR which are designed and synthesized by Sangon Biotech.

*hIL-1β* forward primer, 5′ ATGATGGCTTATTACAGTGGCAA 3′

*hIL-1β* reverse primer, 5′ GTCGGAGATTCGTAGCTGGA 3′

*hTNF α* forward primer, 5′ CCAGACCAAGGTCAACCTCC 3′

*hTNF α* reverse primer, 5′ CAGACTCGGCAAAGTCGAGA 3′

*hGAPDH* forward primer, 5′ ACAACTTTGGTATCGTGGAAGG 3′

*hGAPDH* reverse primer, 5′ GCCATCACGCCACAGTTTC 3′

*STUB1* forward primer, 5′ CTCAAGGAGCAGGGAAACCG 3′

*STUB1* reverse primer, 5′ GGAAGAAGTGCGCCTTCACA 3′

To detect the function of endogenous CHIP, we performed the experiments by knockdown of CHIP using RNA interference. Cells were transfected with CHIP siRNAs using LipoRNAiMAX (Invitrogen) according to the manufacturer’s protocols. The sequences of STUB1 specific siRNAs are designed and synthesized by RiboBio (Guangzhou, China).

#1 SenseSeq: CUGGAACAGUAUCGAGGAATT

AntiSeq: UUCCUCGAUACUGUUCCAGTT

#2 SenseSeq: CAACUUUGGGGAUGAUAUUTT

AntiSeq: AAUAUCAUCCCCAAAGUUGTT

#3 SenseSeq: GGAGAUGGAGAGUUAUGAUTT

AntiSeq: AUCAUAACUCUCCAUCUCCTT

### Immunofluorescence assay

Cells were grown on plates for 12 h, and transfected with targeted plasmids or stimulated with ligands. The cells were fixed with 4% paraformaldehyde for 10 min, and then permeabilized with 0.25% Triton X-100 for 30 min. After washing with PBS, the cells were blocked in 5% fetal goat serum for 1 h, and incubated with primary antibody of p65 for 12 h. The cells were incubated with secondary antibody for 1 h after washing with PBS. At last, nuclear DNA of these cells were stained with DAPI (Sigma) for 5 min. The cells were observed under the fluorescence microscope (Olympus, Tokyo, Japan).

### Statistical analysis

The statistical significance of different groups was determined using an unpaired, two-tailed Student’s *t*-test and ANOVA by the GraphPad Prism 5.0 software. All the experiments were repeated at least three times independently and the differences were considered as statistically significant when *P < 0.05, **P < 0.01, and ***P < 0.001.

## Supplementary information

Attribution of authorship

## Data Availability

The data are availability in this manuscript.
