# Supplementary figures and images for: CHIP promotes the activation of NF-κB signaling through enhancing the K63-linked ubiquitination of TAK1
Source: Cell Death Discov. 2021 Sep 17;7:246. doi: 10.1038/s41420-021-00637-3 (PMC8448743; doi:10.1038/s41420-021-00637-3)

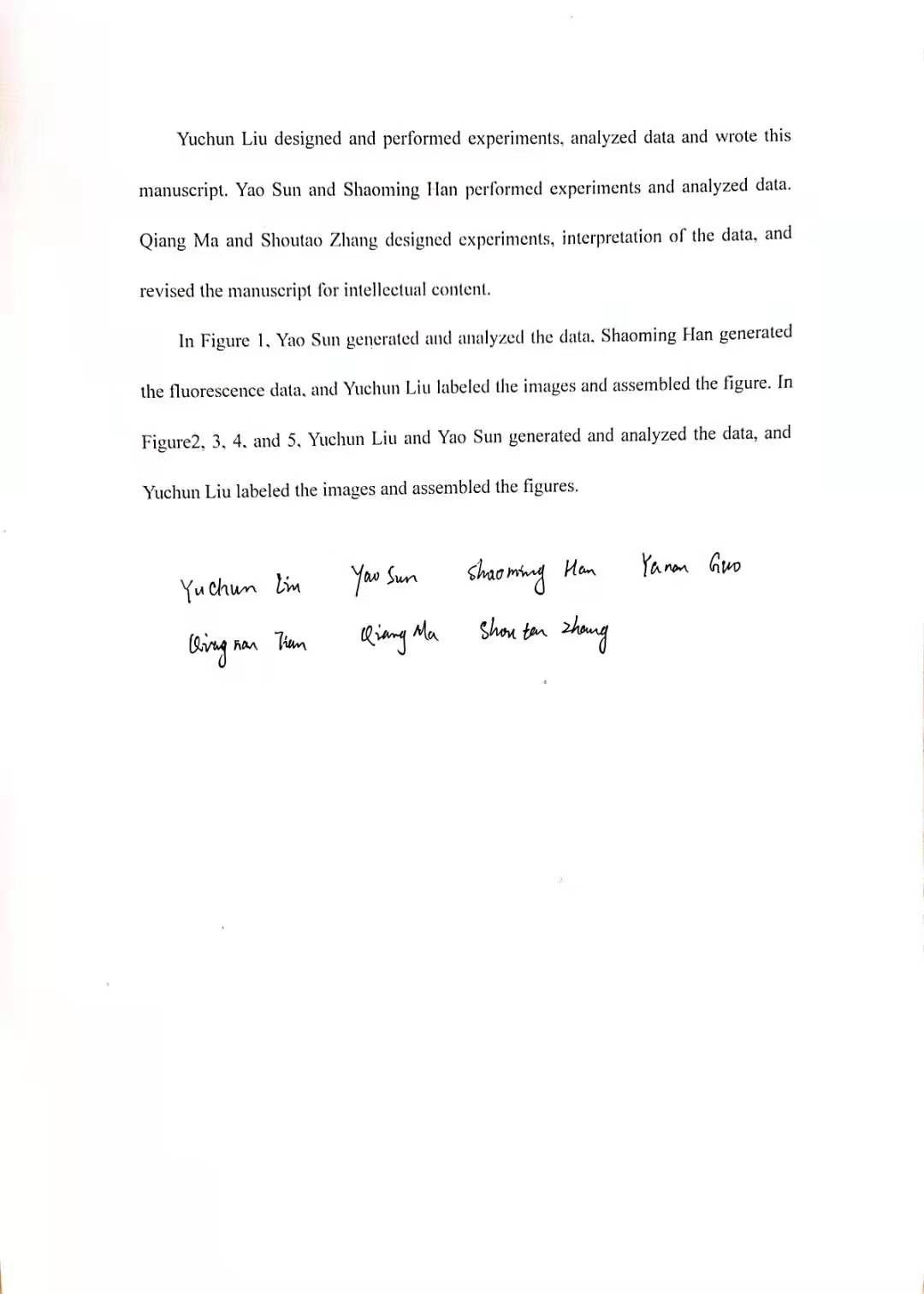

Supplement: Supplementary file 1 — Attribution of authorship [file 41420_2021_637_MOESM1_ESM.jpg]
